# Domestic low‐fat “frying” alternatives: Impact on potatoes composition

**DOI:** 10.1002/fsn3.683

**Published:** 2018-07-03

**Authors:** Carla S. P. Santos, Sara C. Cunha, Susana Casal

**Affiliations:** ^1^ LAVQ@REQUIMTE Laboratory of Bromatology and Hydrology Faculty of Pharmacy University of Porto Porto Portugal; ^2^ ISPUP‐EPIUnit University of Porto Porto Portugal

**Keywords:** acrylamide, antioxidant activity, ascorbic acid, convective oven, fat oxidation, low‐fat frying, microwave grill, sensory analysis

## Abstract

Two low‐fat “frying” alternatives to deep‐frying were tested using two domestic equipment (microwave grill and convective oven), with fresh potatoes and four different frying oils (sunflower, soybean, canola, and olive oil). Potatoes composition was compared concerning nutrients, bioactivity, and fat oxidation. Fat reduction achieved 80% on both methods, directly associated with a decrease in oil natural bioactive components (vitamin E, fatty acids) and degraded lipids (oxidized triglycerides, polymers, aldehydes, etc.). Both microwave grill and oven cooking preserved potatoes and oil health attributes better than deep‐frying, particularly ascorbic acid, tocopherols, and total phenolics. Additionally, a significantly lower formation of acrylamide (−55% microwave grill and −76% oven) and oxidized lipids (oxidized triglycerides and unsaturated aldehydes) was observed, with microwave receiving higher sensory scores than the convective oven. This work sustains the possibility of using domestic equipment (microwave grill and oven) as healthier “frying” alternatives to deep‐frying.

## 1 INTRODUCTION

Potato (*Solanum tuberosum* L.) is one of the most important crops for human consumption, but it needs to be cooked to become edible, mainly due to the presence of toxic glycoalkaloids (Tajner‐Czopek, Rytel, Kita, Pęksa, & Hamouz, 2012). Frying is the most common potato processing technique (Pedreschi, 2012), imposed by operational simplicity, speed, and formation of unique sensorial attributes (Gertz, 2014). However, the inherent incorporation of fat, which can vary from 15% to 45% (Kita, 2014), makes it less advised from the nutritional and health points of view. Some strategies to reduce fat content were studied, including the new air‐frying systems available for domestic cooking (Santos, Cunha, & Casal, 2017; Teruel et al., 2015), or microwave‐assisted vacuum frying with potential industrial applications (Parikh & Takhar, 2016). Still, not all people have access or want to invest in such types of equipment. The use of pre‐fried potatoes on common domestic devices as microwave grill and conventional ovens is also gaining popularity as a reduced fat alternative, but it cannot be regarded as a real low‐fat, with lipid amounts from 7 to 11 g/100 g (Giovanelli, Torri, Sinelli, & Buratti, 2017). Additionally, using pre‐fried potatoes, consumers cannot choose the fat type.

Deep‐frying is based on the direct contact of the food with heated oil, with efficient heat transference by convection (Pedreschi, 2012). Microwave radiation is widely used at a domestic level, enabling short processing times. However, the formation of the typical browning colors is absent, due to the inherent characteristics of the processing principles, based on the interaction of electromagnetic waves with the dielectric properties of the food (Sumnu, 2001). Consequently, the microwave industry developed combined microwave grill/crisp alternatives, capable of generation *Maillard* reactions, now accessible on most domestic microwave devices. Convective oven cooking, on the other hand, uses hot air convection, with some secondary radiation emerging from oven walls and some conduction from the baking pan. Thus, the heat and mass transfer characteristics of foods cooked on these three equipment are entirely different, influencing not only the chemical composition of the processed food but also its sensory properties and hence acceptability.

Based on the abovementioned observations, this work aimed to compare the composition, bioactivity and acceptance, of deep‐fried fresh potatoes with low‐fat domestic alternatives (microwave grill and oven procedures). Aware that the vegetable oil used for cooking has a strong influence on acceptability and composition, we tested four of the most commonly available worldwide (soybean, sunflower, canola, and olive oil).

## 2 MATERIALS AND METHODS

### 2.1 Samples

Potatoes (*Solanum tuberosum* L., Mozart variety) choice was based on their frying aptitude and availability in the local market (Porto, Portugal; potatoes origin: France). Potatoes were manually peeled, cut into 1 cm cubes, washed in plain water, and drained before being processed. No pre‐fried potatoes were used in this study, as the incorporated fat would bias the results. The vegetable oils included commercial soybean oil (SO), sunflower oil (SFO), and olive oil (OO), all available in the local market, and canola oil (CO) acquired from international market (Pforzheim, Germany), with their chemical composition detailed in Supporting Information Table S1.

### 2.2 “Frying” processes

Deep‐frying (DF) was used as control, performed in domestic electric fryers (TRISTAR, FR‐6929 model, The Netherlands, nominal power of 800 W, 1.75 capacity, maximum load 200 g per L). A portion of 200 g of potatoes was fried at 175°C in 1.5 L of fresh oils, during 6 min.

Microwave grill (MWG; Whirlpool, GT285/WH model, USA, nominal power of 1,900 W) and convective oven—OV (Teka, HI‐435 model, Germany, nominal power of 2,565 W) were both carried out using similar potato:oil ratios, using 2.4 g of oil for 200 g of fresh potatoes. The oil was spread homogeneously in the potatoes cubes before processing. The potatoes were cooked in the microwave during 15 min (5 min in microwave function, output power at 700 W; and 10 min in the grill function with the crisp plate, output power at 900 W), manually shaken at 10 and 12.5 min. In the oven, potatoes were cooked for 30 min at 190°C, manually shaken at 15 and 22.5 min potatoes, using a sheet of greaseproof paper commercially available for the purpose.

Each frying process was performed in duplicate, on two different days, using two different bottles of each vegetable oil.

Sensory, physical, and some chemical analyses were performed immediately after cooking, namely moisture and ascorbic acid content. The remaining sample was triturated in an electric grinder (Flama, Portugal), and stored at −20°C until further analyses.

### 2.3 Color

Potatoes color was measured directly on the surface, on six different locations for each frying process and day (*n* = 12 per sample). A Minolta CR‐400 colorimeter (Konica Minolta Optics Inc., Japan) with illuminant D65 was used. The color space system used was CIE‐L*a*b* to represent color coordinate values.

### 2.4 Chemical parameters

The moisture content of cooked potatoes was determined by infrared drying at 105°C (Scaltec SMO 01, Germany). Potatoes lipids were extracted with a ternary mixture of isopropanol, cyclohexane, and water, at room temperature, in the presence of BHT and ascorbic acid, and dried over a nitrogen stream (Santos et al., 2017). Fatty acids composition of both extracted lipids and fresh oils was evaluated by gas chromatography, after cold transesterification with KOH in methanol, according to ISO 12966‐2:^2011^ (2011). Tocopherols were quantified by normal‐phase liquid chromatography with fluorescence detection, as detailed in Santos et al. (2017). Total carotenoids were estimated by UV absorbance, following Nagata and Yamashita (1992).

The antioxidant activity was evaluated in the fresh vegetable oils, after dilution in ethyl acetate, and in the potatoes, after acidic methanol/water extraction followed by acetone/water (Pérez‐Jiménez et al., 2008). Total reducing capacity was determined by the colorimetric Folin–Ciocalteu method while the capacity to scavenge radicals was evaluated by the 2,2‐diphenyl‐1‐picrylhydrazyl (DPPH) radical assay, using gallic acid as the reference on both. Total ascorbic acid was analyzed by HPLC, after dehydroascorbic acid reduction, according to Santos et al. (2017).

Lipid oxidation was evaluated by the *p*‐Anisidine value (*p*‐AV, unidimensional) that measures secondary oxidation products, namely unsaturated aldehydes as 2–alkenals and 2,4–dienals, performed under ISO 6885:^2006^ (2006). Additionally, the total polar compounds (TPC) were quantified by size exclusion chromatography, including oxidized triglycerides (OTG) dimeric and polymeric triglycerides (DPTG), and free fatty acids (FFA), following Dobarganes, Velasco, and Dieffenbacher (2000).

Acrylamide was quantified by GC‐MS, after extraction of potatoes with water and 1,2‐dichloroethane and derivatization with xanthydrol, as detailed in Molina‐García et al. (2015).

All chemical parameters were performed in duplicate for each frying process and day (*n* = 4 per sample).

### 2.5 Sensory analysis

A quantitative descriptive analysis was conducted to characterize and differentiate the sensorial attributes of the fried potatoes in the different oils and for each frying process/day, in a total of 24 samples, using a 10‐cm unstructured linear scale. Ten assessors (8 females and 2 males with age range of 23–55) of the Faculty of Pharmacy of University of Porto were trained for identification of sensory vocabulary and to become familiarized with the scale. Potatoes sensory evaluation was based in nine attributes, describing appearance (color intensity and homogeneity), odor (quality and intensity), taste (quality and aftertaste), and texture (adhesiveness, graininess, and crispiness), in the different oils and for each frying process/day, in a total of 24 samples. The panel was also asked to score global acceptability. In three training pre‐sessions, deep‐fried, microwave and oven‐fried samples were included. The final sensory analyses were performed after 3 min of removing samples from each cooking device. Two evaluation sessions were performed per day, at mid‐morning (around 10:30 hr) and mid‐afternoon (around 16:30 hr). Four samples, marked with three random digits, were presented to the assessors each time, and analyses took place according to Santos et al. (2017).

### 2.6 Statistical analysis

Significant differences, between frying processes or vegetable oils, were analyzed by a one‐way ANOVA (parametric test), using Tukey's post hoc test, or a Kruskal–Wallis (non‐parametric test). These statistical tests were performed using XLSTAT 2016 with statistical significance set at *p* < 0.05.

## 3 RESULTS AND DISCUSSION

Side‐by‐side with the amounts of incorporated lipids, the intrinsic characteristics of each vegetable oil influence the nutritional quality of the final product. On the other hand, the thermal process influences the loss of bioactive potato compounds, as ascorbic acid, together with the formation of new compounds, including lipids oxidation products and acrylamide. A separate discussion of these parameters is detailed below.

### 3.1 Impact on nutritional and health attributes

Moisture and oil contents are among the most important chemical components of fried potatoes, affecting its nutritional and sensory quality (Pedreschi, 2012). Moisture, initially at 81%, decreased with all cooking processes, with reduced but statistically significant differences (*p *<* *0.05) between DF (59%), and both MWG and OV (62%) (Table 1). Regarding incorporated lipids, an average of 6.9% was quantified in DF, against 1.8% in MWG and 1.3% in OV (Table 1), corresponding to 70%–85% reduction of lipids, real low‐fat approaches (<3 g/100 g). These moisture/oil contents are equivalent to those achieved with commercial low‐fat air‐frying systems when using fresh potatoes (Santos et al., 2017) but lower than when using pre‐fried potatoes, with significant amounts of fat already incorporated before processing (Giovanelli et al., 2017). No statistical differences were observed between the different oils tested.

**Table 1:** Effect of different cooking processes and vegetable oils on potatoes composition

		Moistureg/100 g	Lipidsg/100 g	C18:1n‐9 g/100 g	C18:2n‐6 g/100 g	C18:3n‐3 g/100 g	Ascorbic acidmg/100 g	Tocopherolsmg/100 g	Carotenoidsμg/100 g	Total Phenolicsmg GAE/100 g	DPPHmg GAE/100 g
Raw		81.6 ± 1.7	0.14 ± 0.02	–	–	–	6.8 ± 0.1	–	79 ± 13	22.1 ± 0.6	6.1 ± 0.9
Deep‐frying (Control)	SO	59.5 ± 0.2^a,A^	7.0 ± 0.1^a,C^	1.53 ± 0.15^a,C^	2.92 ± 0.15^c,C^	0.32 ± 0.01^b,C^	0.9 ± 0.4^a,A^	4.5 ± 0.3^c,C^	171 ± 23^a,A^	20.5 ± 0.8^b,A^	9.1 ± 0.6^a,AB^
	SFO	58.3 ± 0.4^a,A^	6.7 ± 0.2^a,C^	2.74 ± 0.31^b,B^	2.50 ± 0.30^c,B^	0.03 ± 0.01^a,B^	0.9 ± 0.2^a,A^	3.7 ± 0.3^b,B^	145 ± 14^a,A^	20.6 ± 1.5^b,A^	8.4 ± 0.7^a,B^
	CO	59.0 ± 1.6^a,A^	6.8 ± 0.4^a,C^	3.38 ± 0.03^bc,B^	1.10 ± 0.03^b,B^	0.48 ± 0.02^c,B^	1.2 ± 0.3^a,A^	2.9 ± 0.4^b,C^	167 ± 13^a,B^	13.2 ± 0.4^a,A^	15.5 ± 0.3^b,A^
	OO	60.0 ± 0.3^a,A^	7.1 ± 0.3^a,C^	4.23 ± 0.45^c,C^	0.36 ± 0.03^a,C^	0.04 ± 0.00^a,C^	0.9 ± 0.2^a,A^	0.5 ± 0.1^a,A^	180 ± 36^a,A^	45.4 ± 1.6^c,A^	7.8 ± 0.6^a,B^
Microwave grill	SO	61.4 ± 1.4^a,A^	1.8 ± 0.0^a,B^	0.28 ± 0.03^a,B^	0.56 ± 0.06^c,B^	0.07 ± 0.01^b,B^	1.7 ± 0.6^a,AB^	1.2 ± 0.1^b,B^	177 ± 11^c,A^	29.0 ± 0.8^b.B^	9.3 ± 0.4^b,B^
	SFO	62.5 ± 1.5^a,B^	1.7 ± 0.1^a,B^	0.53 ± 0.03^b,A^	0.51 ± 0.03^bc,A^	0.01 ± 0.00^a,A^	2.4 ± 0.3^a,C^	1.1 ± 0.1^b,A^	130 ± 19^a,A^	26.6 ± 1.5^b,B^	7.2 ± 0.7^a,AB^
	CO	60.0 ± 1.7^a,A^	1.8 ± 0.1^a,B^	0.62 ± 0.06^b,A^	0.33 ± 0.10^ab,A^	0.08 ± 0.04^b,A^	2.2 ± 0.6^a,B^	1.0 ± 0.0^b,B^	143 ± 6^ab,AB^	20.2 ± 0.4^a,B^	15.6 ± 0.3^c,A^
	OO	62.4 ± 0.2^a,AB^	1.8 ± 0.0^a,B^	0.80 ± 0.17^b,B^	0.09 ± 0.01^a,B^	0.02 ± 0.00^a,B^	2.2 ± 0.8^a,B^	0.5 ± 0.0^a,A^	166 ± 3^bc^	54.9 ± 0.9^c,B^	6.6 ± 0.2^a,A^
Oven	SO	63.5 ± 0.6^a,B^	1.3 ± 0.0^a,A^	0.19 ± 0.02^a,A^	0.39 ± 0.04^c,A^	0.05 ± 0.01^b,A^	2.7 ± 0.9^ab,B^	0.8 ± 0.1^b,A^	146 ± 22^ab,A^	27.5 ± 0.8^b,B^	8.5 ± 0.3^b,A^
	SFO	63.3 ± 1.3^a,B^	1.4 ± 0.1^a,A^	0.40 ± 0.09^b,A^	0.39 ± 0.09^c,A^	0.01 ± 0.00^a,A^	1.5 ± 0.3^a,B^	0.8 ± 0.2^b,A^	120 ± 27^a,A^	28.1 ± 1.5^b,B^	6.4 ± 0.8^a,A^
	CO	60.7 ± 0.1^a,A^	1.4 ± 0.1^a,A^	0.50 ± 0.10^b,A^	0.19 ± 0.03^b,A^	0.08 ± 0.02^b,A^	1.5 ± 0.1^a,AB^	0.8 ± 0.1^b,A^	127 ± 8^ab,A^	22.0 ± 0.7^a,C^	16.4 ± 0.4^c,B^
	OO	64.0 ± 2.1^a,B^	1.3 ± 0.0^a,A^	0.51 ± 0.08^b,A^	0.06 ± 0.01^a,A^	0.01 ± 0.00^a,A^	3.2 ± 0.7^b,B^	0.4 ± 0.0^a,A^	157 ± 22^b,A^	56.1 ± 0.8^c,B^	6.4 ± 0.3^a,A^

*Note*. C16:0, palmitic acid; C18:1n‐9, oleic acid; C18:2n‐6, linoleic acid; C18:3n‐3, α‐linolenic acid; CO: canola oil; DPPH: 2,2,diphenyl‐1‐picrylhydrazyl; GAE: gallic acid equivalents; OO: olive oil; SFO: sunflower oil; SO: soybean oil.

^a–d^Statistically significant differences (*p *<* *0.05) between vegetable oils for the same frying process or ^A–C^between frying processes for the same vegetable oil.

When the oils extracted from processed potatoes are compared in terms of fatty acid composition, a dominance of oleic acid in OO fried potatoes, of linolenic acid in SO and SFO, and linolenic acid in CO and SO was observed on all the processes (Table 1). This confirms that, using fresh potatoes, the oils choice are totally reflected in the final product, even in low‐fat approaches, which cannot be granted when using commercial pre‐fried potatoes.

Potatoes are an excellent source of ascorbic acid (Love & Pavek, 2008). However, the thermal and oxidative stress imposed during cooking inevitably induces losses on this vitamin. From an initial amount of 6.8 mg/100 g in raw potatoes, the values decreased significantly, ranging from 0.9 to 3.2 mg/100 g (Table 1). Regarding the frying processes, DF induced an average loss of 85%, significantly higher on all occasions (*p *<* *0.05) than the other two processes, but no significant differences were perceived between the two low‐fat frying processes, with losses ranging from 53% to 78%. Aware that processing occurs faster with DF (6 min) than with the other two alternatives (15 min MWG and 30 min OV), processing time cannot be the primary determinant for ascorbic acid loss, nor moisture, equivalent on all processes. Thermal transfer efficiency, higher in DF, can have contributed to this effect, as well as some potential leaching accompanying water loss in DF. Regarding the different oils, no differences were perceived in the vitamin contents, except an apparent reduced loss with OO in OV, indicative that the potential contribution of the oil components to an oxidant/antioxidant effect is reduced. The ascorbic acid values in this study are lower than those found in fried potatoes (Fillion & Henry, 1998), probably derived from the variety used and the small dimensions of the cubes, necessary to increase homogeneity between processes. Nevertheless, these values are higher than those found in air‐frying systems under similar processing conditions (Santos et al., 2017).

Vitamin E is almost absent in raw potatoes (<0.02 mg/100 g), but present in substantial amounts in the vegetable oils, with intrinsic differences between them (Supporting Information Table S1), therefore enriching potatoes by lipid incorporation (Table 1). Within each cooking process, a higher tocopherol content was quantified in SO, followed by SFO and CO, with the lowest amounts in OO (*p *<* *0.05). Between processes, DF had significantly higher amounts of tocopherols due to the higher lipid content, while the differences between the two low‐fat processes were reduced, imposed by the small amounts of incorporated fat, and similar to air‐frying systems under similar processing conditions (Santos et al., 2017).

Raw potatoes are a recognized source of carotenoids in the diet, particularly β‐carotene, in this case with 79 μg/100 g on a fresh basis (Table 1). The amounts increased on all the processing methods, ranging from 120 μg/100 g (SFO‐OV) to 180 μg/100 g (OO‐DF), influenced by both moisture loss and fat incorporation. However, no differences were perceived between oils (*p *>* *0.05), despite the vegetable oil original composition (Supporting Information Table S1), except for MWG (*p *<* *0.05), with superior amounts with SO and OO. When the processes are compared, no statistical differences were also perceived, except for CO, with higher amounts in DF, followed by MWG and OV, but all within the values presented by the other processes.

Regarding total phenolics, expressed in equivalents of gallic acid (Table 1), raw potatoes amounts (22 mg/100 g) increased with all cooking processes. These could again derive directly from moisture loss, but the oil type had some influence: OO natural richness in phenolic compounds (Supporting Information Table S1) might have induced significantly higher amounts (*p *<* *0.05) on all cooking processes. No differences were perceived between the two low‐fat processes, both presenting significantly higher amounts than DF potatoes (*p *<* *0.05), for all vegetable oils. Aware that fat incorporation was reduced in the low‐fat frying processes, this increase in phenolic content, more than doubling the initial raw potatoes content, could also derive from an increased protection during processing. Moreover, these values are in the same order than those found in air‐frying systems (Santos et al., 2017).

The results for the antioxidant activity under the DPPH radical assay (Table 1) showed that CO imposed consistently higher activity on all cooking processes, as already expected from the oil composition (Supporting Information Table S1), followed by SO, SFO, and OO. Processing differences had a small impact on the final results and, in all cases, the potatoes increased their antioxidant activity.

### 3.2 Impact on lipid degradation and acrylamide formation

During the thermal and oxidative stress induced by cooking, the lipids are gradually deteriorated. Several degradation indicators were used to understand the impact of each cooking procedures on the lipids quality, enabling to compare the potential ingestion of degraded lipids through cooked potatoes. Therefore, the lipids extracted from the processed potatoes were evaluated in terms of classical degradation indexes. It included the *p*‐anisidine value, which gives an estimation of aldehydes formed as secondary oxidation products potentially impacting on odor quality and intensity, together with triglycerides hydrolysis, oxidation, and polymerization (Table 2), presented on a lipid basis to enable a direct association of fat degradation between processes and oils.

**Table 2:** Effect of different cooking processes and vegetable oils on the degradation of incorporated fat

g/100 g extracted lipids		*p*‐AV*	TPC	DPTG	OTG	FFA	TFA
Deep‐frying (Control)	SO	42 ± 8^b,B^	7.6 ± 0.4^b,B^	2.0 ± 0.3^b,C^	3.1 ± 0.2^b,B^	0.5 ± 0.1^a,A^	0.39 ± 0.003^b,B^
	SFO	45 ± 6^b,B^	7.8 ± 0.7^b,B^	2.4 ± 0.4^b,B^	2.9 ± 0.3^b,B^	0.6 ± 0.0^b,A^	0.19 ± 0.002^a,B^
	CO	53 ± 1^b,C^	6.4 ± 0.7^ab,B^	1.7 ± 0.3^b,C^	2.6 ± 0.4^ab,B^	0.7 ± 0.0^b,A^	0.55 ± 0.009^c,B^
	OO	17 ± 2^a,B^	5.9 ± 0.3^a,B^	1.0 ± 0.1^a,B^	2.0 ± 0.2^a,B^	0.5 ± 0.0^a,A^	0.14 ± 0.003^a,B^
Microwave grill	SO	2 ± 1^a,A^	3.9 ± 0.1^c,A^	0.13 ± 0.00^c,B^	1.3 ± 0.0^b,A^	0.5 ± 0.1^a,A^	0.26 ± 0.003^c,A^
	SFO	6 ± 1^b,A^	3.5 ± 0.0^b,A^	0.12 ± 0.01^c,A^	1.1 ± 0.2^b,A^	0.5 ± 0.2^a,A^	0.13 ± 0.002^b,A^
	CO	4 ± 1^a,A^	3.2 ± 0.1^a,A^	0.09 ± 0.01^b,B^	1.1 ± 0.1^b,A^	0.6 ± 0.1^a,A^	0.30 ± 0.007^c,A^
	OO	2 ± 1^a,A^	3.1 ± 0.2^a,A^	0.04 ± 0.01^a,A^	0.7 ± 0.1^a^	0.5 ± 0.0^a,A^	0.08 ± 0.002^a,A^
Oven	SO	2 ± 1^a,A^	3.9 ± 0.0^c,A^	0.10 ± 0.01^b,A^	1.3 ± 0.2^c,A^	0.6 ± 0.1^a,A^	0.21 ± 0.002^c,A^
	SFO	6 ± 1^b,A^	3.2 ± 0.3^ab,A^	0.11 ± 0.02^b.A^	1.0 ± 0.2^b,A^	0.6 ± 0.1^a,A^	0.13 ± 0.001^b,A^
	CO	2 ± 1^a,A^	3.1 ± 0.1^a,A^	0.06 ± 0.01^a,A^	1.0 ± 0.0^b,A^	0.7 ± 0.0^a,A^	0.34 ± 0.004^d,A^
	OO	3 ± 2^ab,A^	3.3 ± 0.1^b,A^	0.05 ± 0.01^a,A^	0.6 ± 0.0^a,A^	0.7 ± 0.1^a,B^	0.06 ± 0.000^a,A^

*Note*. CO: canola oil; DPTG: dimeric and polymeric triglycerides; FFA: free fatty acids; OO: olive oil; OTG: oxidized triglycerides; *p*‐AV: anisidine value; SFO: sunflower oil; SO: soybean oil; TFA: *trans* fatty acids; TPC: total polar compounds.

^a–d^Statistically significant differences (*p *<* *0.05) between vegetable oils for the same frying process or ^A–C^between frying processes for the same vegetable oil; *expressed as 100 times the optical density measured at 350 nm (1 cm) of a solution containing 1.00 g of the oil in 100 ml, according to the method.

Regarding oxidation, *p*‐AV values were consistently higher in DF, without differences between the two low‐fat processes. When the oils are compared within each process, OO had significantly lower *p*‐AV values, being, therefore, less oxidized, which could be a consequence of its higher phenolic content although its lower polyunsaturated degree also contributes for its stability. In the low‐fat processes, the *p*‐AV value was generally low, reducing the ability to distinguish the different oils, but SFO had higher amounts of secondary aldehydes.

The TPC are a consensual index for oil degradation, corresponding to the sum of OTG with polymeric forms (DPTG), diglycerides (DG) and free fatty acids (FFA), with 22%–25% as the limit for rejection in several European countries (Gertz, 2000). For the polymeric forms, DF potatoes had significantly higher amounts (5.9%–7.8%), being only residual in the two low‐fat approaches (3.1–3.9), without differences between them (Table 2). The oxidized triglycerides (OTG), again higher in DF, enabled a distinction between the low‐fat processes, with higher amounts in MWG than OV for SO and CO.

Fatty acids isomerization is also an indicator of thermal and oxidative degradation although occurring at relatively low rates and amounts (Table 2). Indeed, TFA were low with all extracted lipids, slightly higher in CO, and similar to those of the fresh oils (Supporting Information Table S1). As to the processes, higher TFA were quantified in the DF, but always below 0.05%, corresponding to low amounts from a health point of view.

Acrylamide was included in this section because it corresponds to a compound formed during heating, with potentially deleterious effects on consumer's health. Acrylamide is a recognized processing contaminant in fried potatoes, being formed above 120°C and under low‐moisture conditions (Molina‐García et al., 2015). Mean values from 130 (OV) to 550 μg/kg (DF) were detected in the cooked samples, with a clear distinction between processes. Deep‐fried potatoes presented the highest amounts (*p *<* *0.05) (718‐SFO, 606‐SO, 510‐OO, 369‐CO, in μg/kg), followed by MWG (298‐CO, 280‐SFO, 248‐OO, 186‐SO, in μg/kg) and OV [207‐SO, 144‐CO, 95‐SFO, 75‐OO, in μg/kg). The higher amounts in MWG compared to OV can probably be explained by the direct contact with the crisp plate in the former, with probable increased *Maillard* reactions extension on the darker spots perceived although the influence of microwaves themselves is not excluded (Ye, Miao, Zhao, & Yuan, 2011). This is also consistent with the increased red tones (a*) in the instrumental color reading (*p *<* *0.05) [DF (3) > MWG (−2) > OV (−4)] and yellowness (b*) [DF (29) > MWG (26) > OV (24)] while lightness (L*) was similar on all processing conditions. The average scores for each color coordinate were in agreement with the recommended tones for fried potatoes: between −5 and 0 for a* and > 10 for b* (Krokida, Oreopoulou, Maroulis, & Marinos‐Kouris, 2001) and similar to those reported for air‐frying systems (Santos et al., 2017; Teruel et al., 2015). When oils are compared, a clear pattern was not perceived, with SFO presenting simultaneously the highest average amounts in DF and MWG, with 718 μg/kg and 280 μg/kg, respectively, and being within the lowest range in OV, with 95 μg/kg. In opposition, CO presented the lower acrylamide content in DF (369 μg/kg) but was within the highest range in MWG (298 μg/kg). This high variability between oils shows that the potential effects imposed by the processing technique superimpose those potentially derived from the oils composition.

These acrylamide content reduction reductions observed with MWG and OV are interesting from a health point of view and they represent a 70% reduction when compared with compared with air‐frying systems under similar processing conditions (640 μg/kg, on average) (Santos et al., 2017).

### 3.3 Impact on sensorial quality

**Figure 1:**
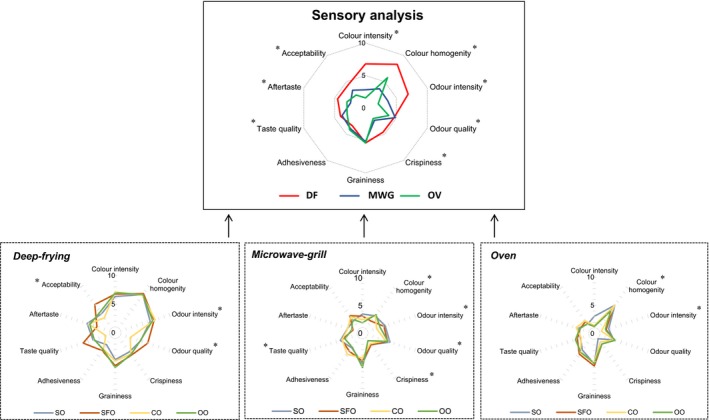
Sensory analysis of fried potatoes (* for significant differences (*p *<* *0.05) between frying processes and frying oils)

Independently of the oil type (Figure 1), most sensorial attributes presented higher scores for classical deep‐frying (*p *<* *0.05): color and odor intensities (DF > MWG > OV), color homogeneity (DF > OV > MWG), odor quality (DF, MWG > OV), crispness and aftertaste (DF > MWG, OV), and taste quality (DF, MWG > DF, OV). Adhesively and graininess were similar between processes (*p *>* *0.05). The lower crispness scores on both low‐fat approaches are directly associated with water loss and fat incorporation data, previously discussed, essential for crust formation (Melema, 2003). Data also show that, even with frequent turnover, oven and microwave grill are unable to achieve DF color homogeneity due to the direct contact with the metal recipient surfaces. Still, when MWG and OV are compared, MWG presented better odor and color intensities, as well as taste and color qualities, but lower color homogeneity, in agreement with the instrumental color measures. Crispiness and aftertaste were also similar on both low‐fat approaches. Global acceptability corroborated the individual scores, with higher acceptability for DF (4.5), followed by MWG (3.4) and OV (2.5). The MWG scores were within the range of commercial air‐frying devices (3.5–4.3) tested under similar processing condition (Santos et al., 2017). When the different oils were compared under DF, a general preference for SFO was perceived (6.1/10 global mean score) in opposition to CO (3.2/10), but no significant differences were observed in the low‐fat processes, probably due to the low amounts of incorporated fat (Figure 1).

## 4 CONCLUSION

The results of this work demonstrated that MWG and OV have potential to be implemented at domestic level as healthier “frying” alternatives. Its use with fresh potatoes, instead of pre‐fried frozen ones, enables consumer to choose the oil of their preference while achieving true low‐fat potatoes. Indeed, the amount of fat absorbed by the MWG and OV potatoes is around 80% lower than DF, corresponding to an average decrease in 50 kcal/100 g under the present conditions. However, the advantages are not restricted to the lipid amounts, with direct ingestion of fewer amounts of degraded fat, higher preservation of potatoes ascorbic acid, and total phenolic compounds, particularly with OO, and smaller amounts of acrylamide. Despite the expected higher acceptability for DF potatoes, and the vegetable oil type influence, MWG was sensory preferred to OV, being both similar from the chemical and nutritional points of view. Optimized instructions could be included in the equipment's manuals to grant good cooking results.

## CONFLICT OF INTEREST

The authors declare no conflict of interest.

## ETHICAL STATEMENT

No human or animal testing was involved in this study**.**
