# Supplementary material for: Domestic low‐fat “frying” alternatives: Impact on potatoes composition
Source: Food Sci Nutr. 2018 Jul 3;6(6):1519–26. doi: 10.1002/fsn3.683 (PMC6145265; doi:10.1002/fsn3.683)
Supplement: Supplementary file 1 [file FSN3-6-1519-s001.docx]

**Table 1S.** Main characteristics of the fresh vegetable oils used.

| Oil types | C16:0  g/100 g | C18:1n-9  g/100 g | C18:2n-6 g/100 g | C18:3n-3 g/100 g | TFA g/100g | Tocopherols mg/kg | Carotenoids µg/100 g | Total Phenolics mg GAE/kg | DPPH  mg GAE/kg | *p*-AV | TPC  g/100 g | DPTG  g/100 g | OTG  g/100 g | FFA  g/ 100g |
| --- | --- | --- | --- | --- | --- | --- | --- | --- | --- | --- | --- | --- | --- | --- |
| SO | 11.8±0.0 | 25.3±0.2 | 50.0±0.2 | 5.6±0.0 | 0.4±0.0 | 707±40 | 25±3 | 62±3 | 76±2 | 0.6±0.1 | 5.1±0.6 | 0.1±0.0 | 2.4±0.5 | 0.5±0.0 |
| SFO | 6.1±0.1 | 45.6±0.8 | 41.8±0.9 | 0.1±0.0 | 0.2±0.0 | 670±13 | 30±4 | 55±1 | 74±2 | 3.2±0.2 | 3.6±0.2 | 0.1±0.0 | 1.1±0.1 | 0.6±0.0 |
| CO | 4.7±0.3 | 57.7±0.4 | 19.0±0.0 | 8.8±0.2 | 0.4±0.2 | 1156±89 | 19±3 | 64±3 | 82±2 | 0.9±0.1 | 3.2±0.1 | 0.1±0.0 | 1.0±0.0 | 0.7±0.0 |
| OO | 11.8 ±0.2 | 73.4±0.3 | 6.2±0.2 | 0.7±0.0 | 0.1±0.0 | 293±25 | 480±6 | 138±2 | 73±1 | 3.3±0.3 | 4.2±0.2 | 0.1±0.0 | 1.2±0.3 | 0.5±0.0 |
| SO – soybean oil; SFO - sunflower oil; CO - canola oil, OO – olive oil; C16:0 – palmitic acid; C18:1n-9 – oleic acid; C18:2n-6 – linoleic acid; C18:3n-3 – α-linolenic acid; TFA – trans fatty acids; GAE – gallic acid equivalentes; DPPH – 2,2,diphenyl-1-picrylhydrazyl; *p*-AV – *p*-anisidine value; TPC – total polar compounds; DPTG – dimeric and polymeric triglycerides; OTG – oxidized triglycerides; FFA – free fatty acids. | | | | | | | | | | | | | | |
